# 

**Published:** 1880-06

**Authors:** 


					﻿Number,
CCCCXXI.
June, 1880.
Number 6,
Vol. XL.
THE
CHICAGO
MEDICAI
T ournal and Examiner
EDITORS:
N. S. DAVIS, M.D., LL.D. JAS. NEVINS HYDE, A.M., M.D.
DANIEL R. BROWER, M.D.
CHICAGO:
PUBLISHED BY THE CHICAGO MEDICAL PRESS ASSOCIATION,
188 Clark Street.
Jg-Read the Notice of this Journal among Advertisements.
				

